# Supplementary figures and images for: What do we know about the fossil record of pinnipeds? A historiographical investigation
Source: R Soc Open Sci. 2019 Nov 27;6(11):191394. doi: 10.1098/rsos.191394 (PMC6894555; doi:10.1098/rsos.191394)

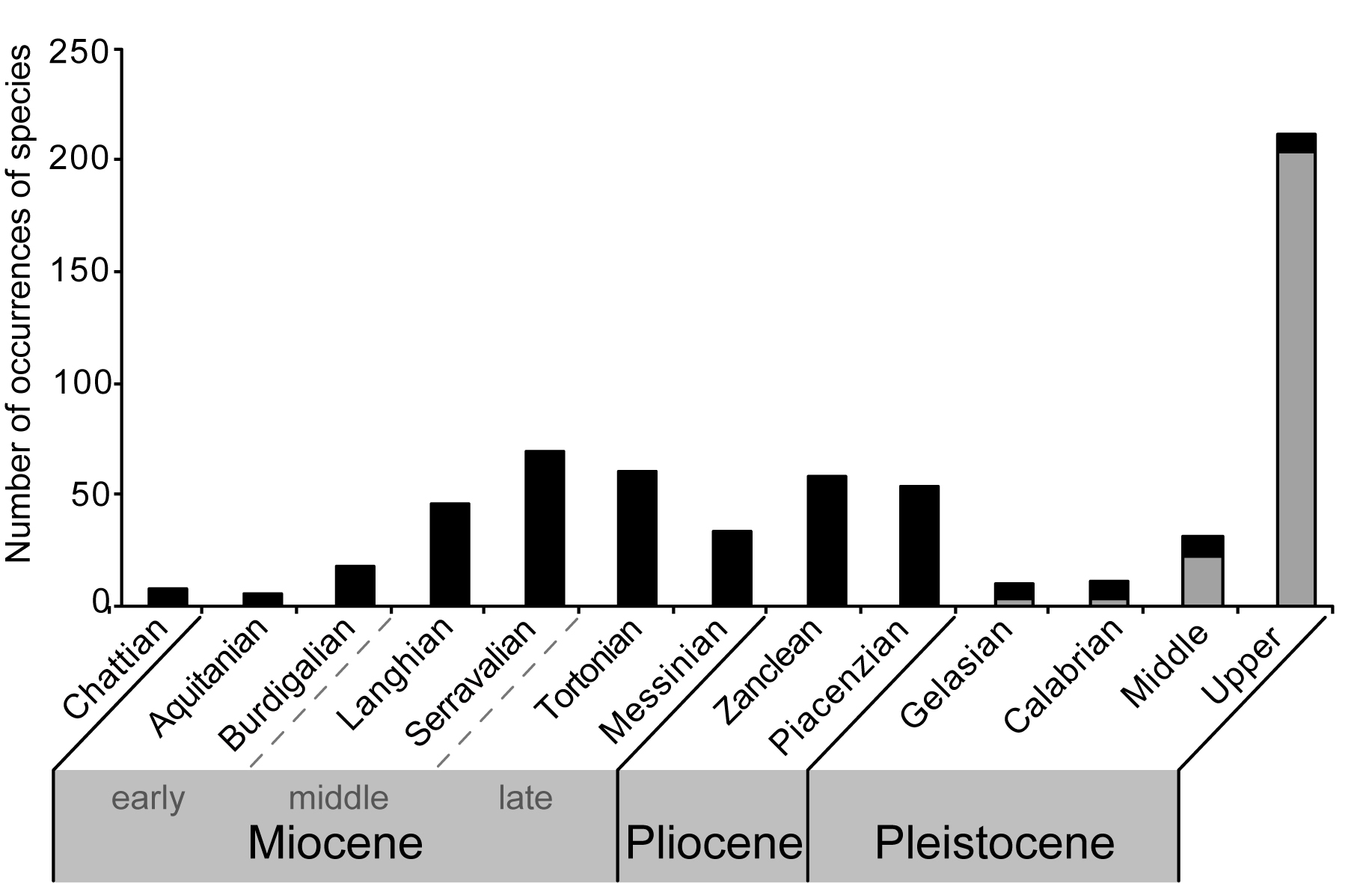

Supplement: SUPPLEMENTARY FIGURE 1 [file rsos191394supp1.jpg]

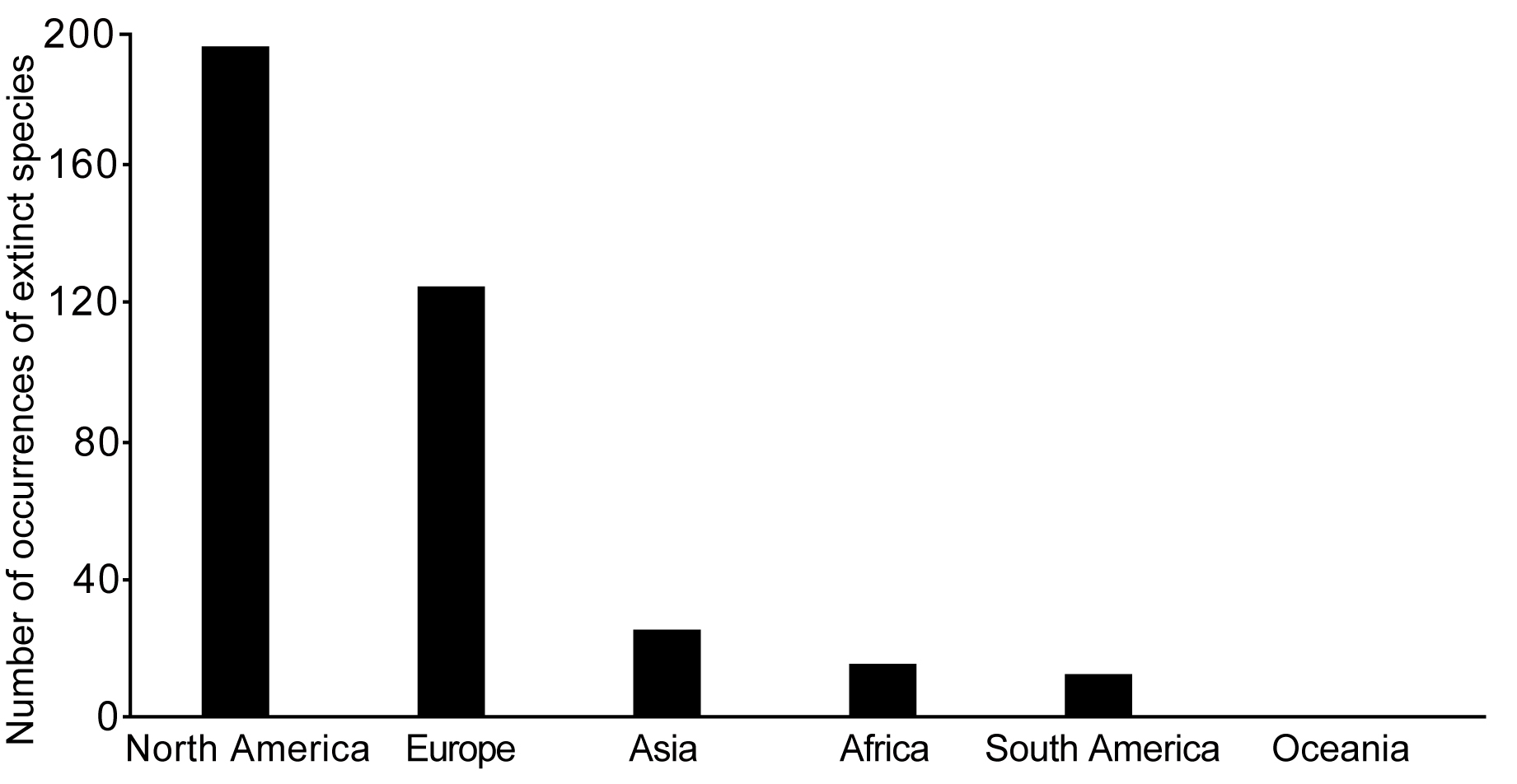

Supplement: SUPPLEMENTARY FIGURE 2 [file rsos191394supp2.jpg]

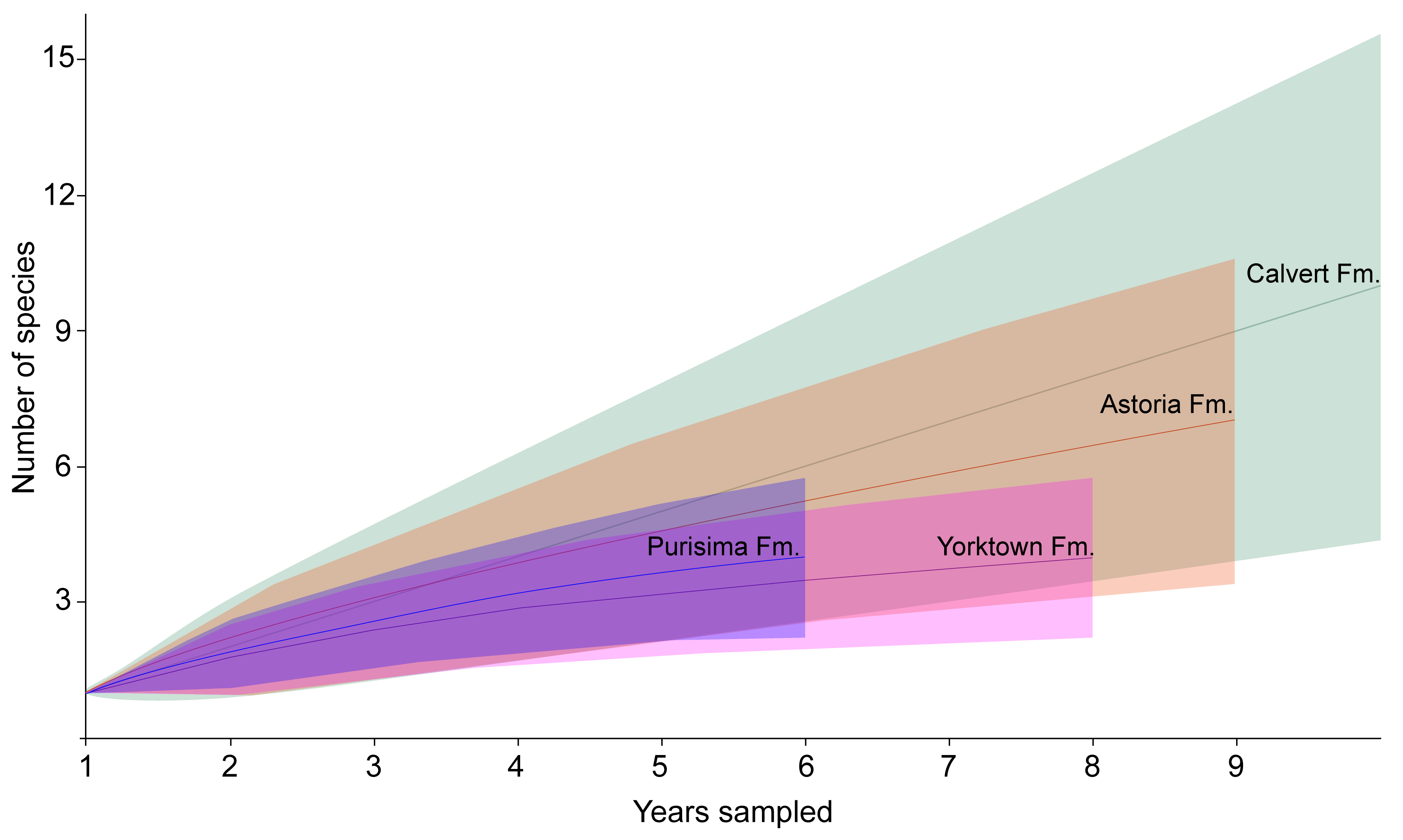

Supplement: SUPPLEMENTARY FIGURE 3 [file rsos191394supp3.jpg]

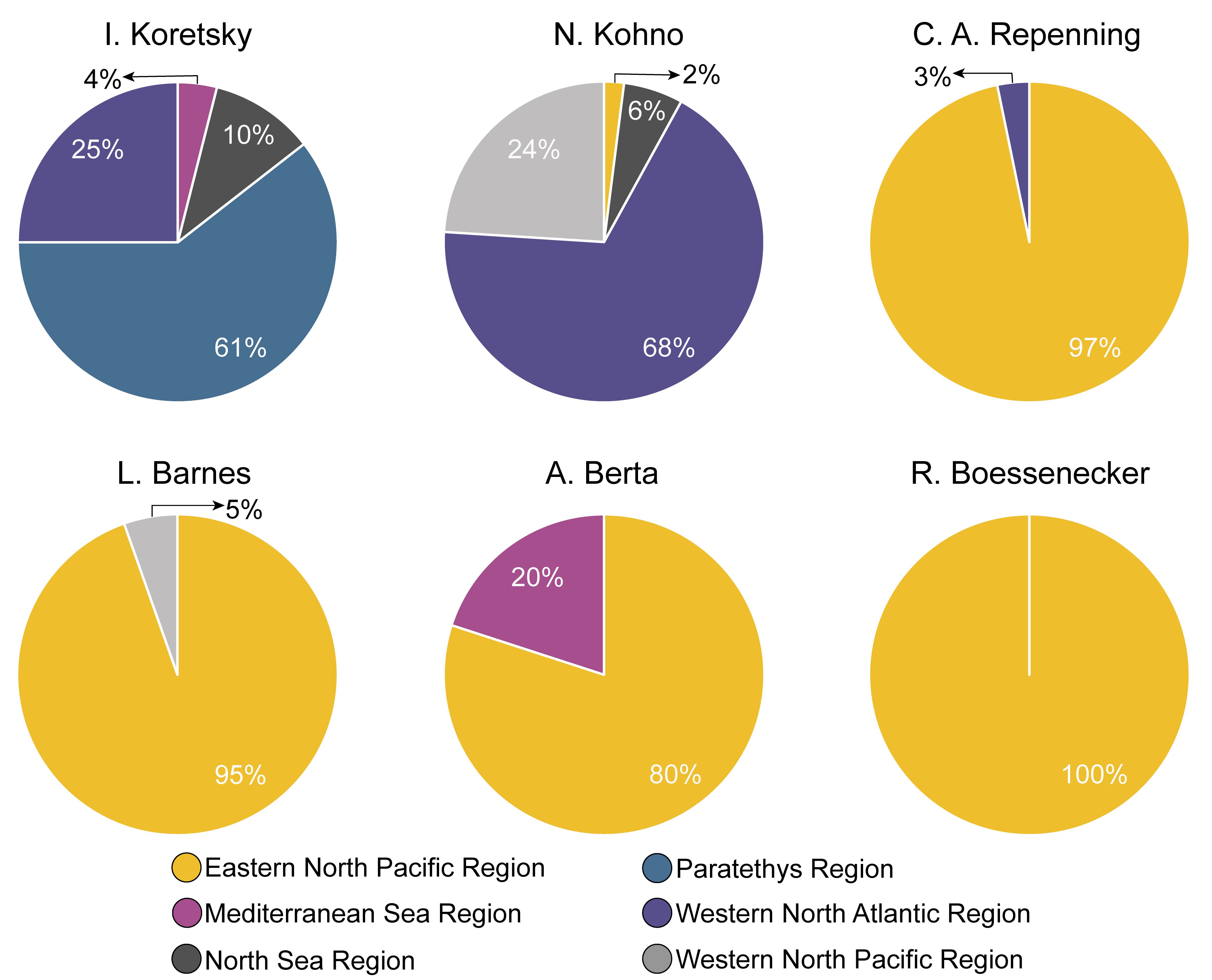

Supplement: SUPPLEMENTARY FIGURE 4 [file rsos191394supp4.jpg]
